# Impact of immediately loaded implant-supported maxillary full-arch dental prostheses: a systematic review

**DOI:** 10.1590/1678-7757-2018-0600

**Published:** 2019-07-25

**Authors:** Ahlam ABDUNABI, Martin MORRIS, Samer Abi NADER, Raphael F. de SOUZA

**Affiliations:** 1 McGill University McGill University Faculty of Dentistry, Oral Health and Society Montreal Canada McGill University, Faculty of Dentistry, Oral Health and Society, Montreal, Canada.; 2 McGill University McGill University Schulich Library Montreal Canada McGill University, Schulich Library, Montreal, Canada.; 3 McGill University McGill University Faculty of Dentistry Montreal Canada McGill University, Faculty of Dentistry, Restorative Dentistry, Montreal, Canada.

**Keywords:** Complete denture, Dental implant loading, Dental implantation, Health-related quality of life, Treatment outcome

## Abstract

The immediate loading of implant-assisted fixed prostheses in edentulous maxillae may achieve favorable success rates with reduced treatment time. An evidence summary of clinical trials is key to recommend loading protocols in these cases.

**Objectives:**

To compare immediately loaded, fully implant-supported complete dentures to early and conventional/delayed loading in the edentulous maxillae of adult patients by a systematic review of controlled clinical trials (CCT).

**Methodology:**

CCTs reports were identified up to January 17, 2019 from Cochrane Oral Health Group’s Trial register, Cochrane Central Register of controlled trials (CENTRAL), MEDLINE (Ovid), BIOSIS, EMBASE, CINAHL, Web of Science, and DARE. Two independent reviewers screened titles/abstracts and confirmed inclusion using full texts. Data were extracted and quality assessed (Cochrane Risk of Bias tool) independently and in duplicate. Study heterogeneity prevented pooling by meta-analysis.

**Results:**

Out of 1,052 candidate studies, four CCTs were included. Two trials had patient satisfaction as an outcome: (1) A randomized trial compared immediately and early loaded fixed dentures and found more satisfaction with the first after 12 months; (2) A non-randomized study found better satisfaction with immediate fixed dentures compared to conventional loading after 3 months (no more at 12 months). Regarding implant success and prosthetic complications, three trials did not report significant differences comparing immediate loading to other protocols.

**Conclusions:**

This review found weak evidence of differences between immediate load and other loading regimens, regarding patient satisfaction and maintenance events/adversities. The potential of immediate loading for favorable results in edentulous maxillae reinforces the need for well-designed RCTs, for solid clinical guidelines. Registration number CRD42018071316 (PROSPERO database).

## Introduction

Edentulism poses a major impact on oral and general health, and on quality of life. Edentulous individuals have higher risk of systemic diseases, as pinpointed by the increased mortality rate among the edentulous elderly.^1 , 2^ Impaired mastication represents a major consequence of edentulism. Even with good-quality complete dentures, masticatory performance is from 1/5 to 30% of dentate patients.^3 , 4^ Besides mastication, conventional denture wearing represents a major psychological and social burden for some patients.^5^

Implant-assisted prostheses can tackle most of the limitations of conventional dentures, regardless of being fixed or removable. Fixed complete prostheses lead to better patient satisfaction in many cases compared to removable alternatives. This is the case when ease of hygiene is not a patient-perceived priority, which is common amongst middle-aged patients.^6^ Primary indications for fixed prostheses include patients who cannot endure removable dentures or the feeling of being edentulous, strong gag reflex, and history of recurrent sore spots caused by dentures.^7^ Patients with previous negative denture experience tend to perceive implant-supported fixed prostheses as their own natural teeth, leading to good self-esteem, physical and social well-being.^5^

Despite the focus given to the lower arch,^8^ many edentulous patients request conversion of their maxillary dentures to implant-assisted ones. The maxillary arch poses specific challenges, including low-density bone^9^ and limiting sinus anatomy.^10^ Furthermore, fixed prostheses are a more intuitive choice for edentulous maxillae, given that patient satisfaction does not seem to improve much with maxillary overdentures.^11^

The possibility of immediate load after implant insertion can expedite an otherwise time-consuming treatment, i.e. maxillary fixed dentures. Studies have demonstrated high success rates for immediately loaded fixed prostheses in edentulous maxillae, with conventional or zygomatic implants.^10 , 12^ Immediate prostheses may be more satisfying for patients than those fabricated by traditional protocols.^13 , 14^

In 2013, a systematic review of randomized clinical trials (RCT) on immediately loaded implants showed no evidence of different success rates when compared to other loading protocols.^15^ This review, despite its high quality, did not approach patient-reported outcomes (PRO) (e.g. satisfaction and oral health-related quality of life). Actually, PRO can be considered the main success indicator for prosthodontics.^16^ Understanding how patients respond to different loading protocols in the edentulous maxilla is essential for developing clinical guidelines. However, there are no systematic reviews considering PROs to understand the effect of these protocols, which would be of primary relevance for clinical recommendations.^17^

Therefore, we present a systematic review of controlled clinical trials (CCT) comparing immediate versus early/delayed loading on implant-supported maxillary complete dentures, in terms of PROs and maintenance events/complications. This review was based on the following PICO question: in maxillary edentulous adults (P), is immediate loading (I) more effective than other loading protocols for full implant-supported prostheses (C) from the patient’s perspective (O)? To reach a broad range of studies, we expanded this question to any treatment modality with complete implant support (i.e. fixed or removable).

## Methodology

This review was reported according to the PRISMA guidelines (checklist available on Appendix 1 ).^18^ A protocol version was published at the PROSPERO database (ID: CRD42018071316).^19^

### Eligibility criteria

Included studies should comply with the following criteria, grouped by design, participants, interventions, comparators and outcomes:

Study design: experimental studies in humans comparing immediate loading to a control group (other loading protocols). The allocation of participants to one of the groups could be random (i.e. RCT) or not (non-randomized CCT). Other designs (e.g. observational studies, one-arm trials) were not eligible.

Participants: Adult patients with edentulous maxillae seeking implant-supported complete dentures.

Interventions: Immediate-loaded, fully implant-supported complete dentures (IL): denture delivery until the 7^th^ day following implant insertion,^20^ regardless of being the final or interim restoration. Dentures should be fixed or removable; in the latter case, they should receive complete support from implants. Eligible removable protocols include milled bars or telescopic attachment, given that the mucosa does not provide retention, stability or support.

Comparators: Similar to the intervention, but with later delivery of a maxillary denture. Comparators were divided into (1) Early Loading (EL): loading between a week and two months after implant insertion; and (2) Conventional Loading (CL, also dubbed delayed loading): loading after more than two months after implant insertion.

Outcome measures: Primary outcomes: general patient satisfaction with prostheses and oral health-related quality of life (OHRQoL), the most common PRO of studies on prosthodontics.^16^ Patient satisfaction could be graded by specific questions answered on categorical or quantitative scales; OHRQoL should be tested by validated questionnaires, including: Oral Health Impact Profile (OHIP), Oral Impacts on Daily Performance (OIDP), Geriatric Oral Health Assessment (GOHAI), and Dental Impact on Daily Living (DIDL), as well as their abbreviated versions.

Secondary outcomes: (1) Specific patient satisfaction items, such as ease in chewing, swallowing, satisfaction with esthetics, and ease of hygiene; (2) Clinician-assessed implant-related parameters: implant success rate, marginal bone level, occurrence of mucositis and peri-implantitis, bleeding on probing (BOP), plaque index and probing depth. (3) Clinician-assessed performance of prostheses: success and survival rates, functional parameters like masticatory performance, technical complications like occlusal wear, screw loosening or fractured prosthetic components.

Due to the short-term response linked to IL and expected longevity of implant-assisted prostheses, we did not consider a particular timespan. We sought to discuss results for primary outcomes based on short-term results whenever possible, i.e. within the first three months after loading.

### Search Methods

MM, a librarian trained in systematic review searching, conducted an electronic search in MEDLINE (Ovid), PubMed, EMBASE (Ovid), BIOSIS (Ovid), Cochrane Oral Health Group’s Trial register; Cochrane CENTRAL and DARE databases (the Cochrane Library 2019, issue 1), CINAHL; and Web of Science. Searches were performed on July 14, 2016, and update searches were performed on May 22, 2018 and January 17, 2019; results were limited to researches from 1999 onwards, due to the effective introduction of IL in the 1990s. Appendix 2 shows the search strategy used for MEDLINE via Ovid, which was adapted for each database. Given the search yield, we did not apply any filter or outcome-specific term. We also screened the list of references of included studies and reviews on immediate loading. The search was restricted to articles in English.

Two authors (AA and RFS) scanned the titles and abstracts of all reports identified through the electronic searches independently. A 3^rd^ reviewer (SAN) was contacted as required to resolve disagreements. The same authors examined full-text versions of possible inclusions independently.

### Data extraction and quality assessment

Included studies underwent data extraction and quality assessment by the same authors. We extracted data from trials based on the following characteristics:

1. Study design: time until follow-up, sample size, study setting, sampling criteria, recruitment methods, randomization methods, randomized number, drop-outs, withdrawals and losses;
2. Participant: age, gender, general health status (including diabetes mellitus), clinical characteristics (history of periodontitis, maxillary bone volume and density), smoking, drinking habits, other recreational drugs, occlusion during healing phase, previous experience with removable dentures, and attendance to follow-up visits;
3. Intervention and comparators: implants (system, number, type, design, length, positioning, and insertion torque), interim prosthetic design and loading time (if applicable), and definitive prosthetic design and loading time;
4. Outcomes: Assessment method and instrument, baseline and post-treatment scores, as well as time of data collection.

We assessed the quality of included trials by using the Cochrane Risk of Bias tool.^21 , 22^ This classifies studies based on six potential sources of bias: (1) random sequence generation (selection bias), (2) allocation concealment (selection bias), (3) blinding (performance bias and detection bias), (4) incomplete outcome data (attrition bias), and (5) selective reporting (reporting bias), as well as (6) other sources. Each potential sources was classified as low, unclear or high. Moreover, the tool allows an overall classification of study risk of bias, i.e. any high-risk source renders the study as high risk of bias, whereas low-risk studies have all sources classified as such. Studies with any unclear source but no high-risk source were classified as moderate risk of bias.

### Summary measures and statistical analysis

Most patient satisfaction and OHRQoL-related variables are continuous, and thus could be described according to their mean differences and 95% confidence interval (95%CI). Those included items answered on visual analogue scales (VAS) and summed results from Likert/ordinal scales. Similar strategies were used for other quantitative outcomes, including bone level changes. Dichotomous variables (e.g., frequency of prosthesis fracture, or occlusal wear: Yes/No) were described according to risk ratios (RR) with 95%CI. Whenever there were some issue regarding the unit of analysis for dichotomous variables (two or more event counts for the same participant), data was shown as cumulative incidence only. Inferences based on a 95%CI correspond to the adoption of a level of significance (α) of 0.05. The RevMan 5.3 software was used for plotting quality assessment and effect measures.

If two or more trials reporting the same comparison and outcome were found, we would assess their heterogeneity. In turn, we would synthetize data by meta-analysis if applicable, giving priority to random effect models. We also planned to assess publication bias using a funnel plot, if there were sufficient studies. Please refer to our review protocol for details on planned statistical methods.^23^

## Results

### Search results

**Figure 1:**
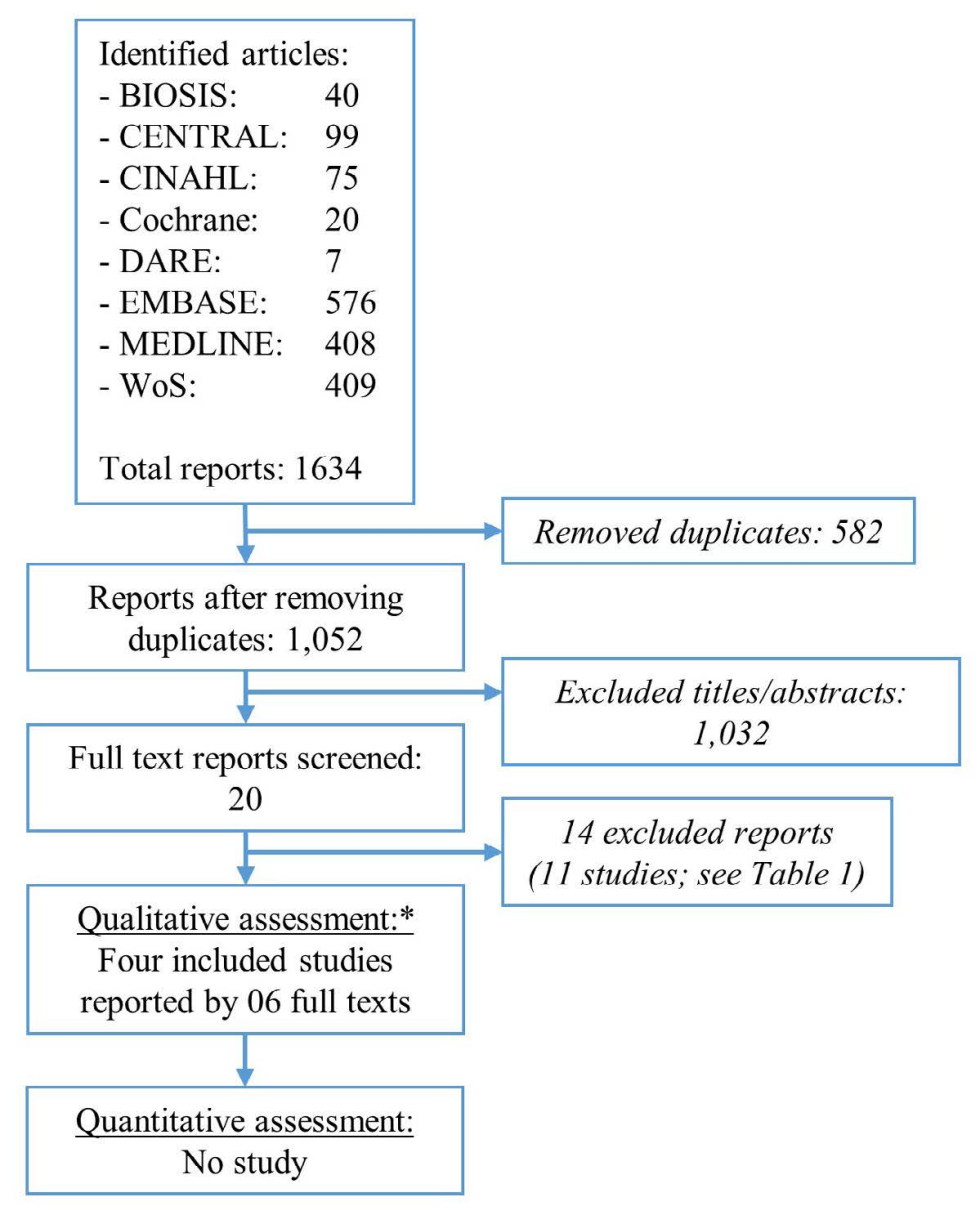
Flow diagram of study selection. No study was located from other sources, including the references of screened full text reports and reviews. *Qualitative appraisal of the body of studies (without quantitative synthesis/meta-analysis)

Figure 1 summarizes the search yield and study selection. We identified 1,052 reports by the electronic searches (duplicates excluded). Reading of titles and abstracts led to the exclusion of 98.1%, and to further appraisal of 20 full-text versions (1.9%). In turn, we included four trials reported by six manuscripts (two trials had their results published in two manuscripts each). Two of such trials provided data on patient satisfaction with received prostheses (primary outcome), whereas none assessed OHRQoL.

Eleven studies were excluded ( Appendix 3 ).^24 - 37^ Reasons included ineligible study designs (five studies) and ineligible comparator groups (five studies). A study dealt with partial edentulism, an RCT had a mixed sample with partial and complete edentulism (both arches), and another evaluated a non-eligible intervention. ^24 - 29 - 30 - 33 - 34 - 37^

### Characteristics of included studies

The two trials evaluating patient satisfaction assessed 59 participants, with a single loss ( Table 1 ).^13 , 14 , 38^ Both applied nearly similar inclusion and exclusion criteria on the initial samples of 30 participants/each: edentulous maxillary arches with existing opposing occlusion, not needing augmentation procedures. Lower arches had natural teeth (complete dentition or combined with dental prostheses) or implant-assisted prosthesis. The RCT by Canizzaro, et al.^38^ (2008) recruited patients at an Italian private clinic from 2004 to 2005 to compare IL to EL. Follow-up extended to 12 months.^38^ The non-randomized CCT by Penñarrocha-Oltra, et al.^13 , 14^ (2013, 2014) compared IL to CL.^13 , 14^ Researchers enrolled participants at a Spanish university clinic from 2008 to 2010, treated according to patient preferences. Both studies provided provisional acrylic maxillary fixed dentures immediately after implant insertion for IL. Provisional dentures were replaced by porcelain-fused-to metal (PFM) or metal-resin bridges after nearly 3 months.

**Table 1:** Summary of the included study characteristics

Study ID	Cannizzaro, et al.^**38**^ (2008)	Peñarrocha-Oltra, et al.^**13,14**^ (2013, 2014)	Tealdo, et al.^**39,40**^ (2011, 2014)	Vercruyssen, et al.^**41**^ (2016)
**Sample size, n participants**	Initial : 30. IL: 15 (90 implants); EL: 15 (87 implants)	Initial: 30. IL: 15 (94 implants). CL: 15 (99 implants). IL: 1 loss	Initial: 49. IL: 34 (163 implants). CL: 15 (97 implants). 1 loss/group	Initial: 15. IL: 7 (42 implants). CL: 8 (48 implants).
**Outcome variable and instrument**	(1) Overall patient satisfaction on 5-point Likert scale (2) Frequency, clinical complications: damaged prostheses, peri-implant adversities, lost implants, mucosal lesions	(1) Overall patient satisfaction (100-mm VAS) (2) Patient appraisal of aesthetics, chewing, speech, comfort, self-esteem and hygiene (100-mm VAS)	(1) prosthodontic survival (3) marginal bone level (4) prosthetic complications	Short-term implant failure rate
**Data collection timeline**	Up to 12 months. Patient satisfaction collected at 12-mo. (no baseline data)	Baseline, 3-mo. ,and 12-mo. follow-up	Baseline,1-, 2-, 3-, and 6-y follow-up	Up to 3 months
**Implants: n, insertion torque**	n (patients): 5 (IL: 5, EL: 7); 6 (IL: 6, EL : 5); 7 (IL: 3, EL: 2) ; 8(1/arm). Torque >48 Ncm	n: 6-8 per patient. Torque >35 Ncm	n IL, mean: 4.6; range: 4 to 8. N CL: mean: 6.5 range: 6 to 9. Torque ≥40 Ncm	6 per patient. No data on torque
**Implant system**	Tapered Swiss Plus (Zimmer Dental, Carlsbad, CA, USA); diameter: 3.7 to 4.8 mm; length: 10, 12 and 14 mm	Kohno SP (Sweden & Martina SpA, Padova, Italy)	Osseotite and Osseotite NT (Biomet 3i); diameter: 4 mm	Ankylos (Dentsply Implants, Molndal, Sweden); diameter: 3.5 or 4.5 mm; length: 9.5 to 14 mm

The other two included studies restricted their outcome assessment to clinical variables, and compared IL to CL. Both were conducted at university clinics and included further 64 participants (1 lost participant/arm). A non-randomized CCT in Italy compared IL on 4 to 6 implants to CL on 6 to 9 implants (loading time; IL: ≤24 h; CL: ~9 mo.).^39 , 40^ Recruitment happened between September 2005 and January 2006. Participants in the IL arm wore a transitional screw-retained acrylic fixed denture with a cast metal framework and without cantilevers during 4.5 months, followed by the definitive prostheses. Both arms received similar acrylic screw-retained definitive prostheses with one-tooth long cantilevers and cast metal frameworks. An RCT in Belgium also compared IL to CL (24 h versus 3 months) on a non-variable number of six implants (surgery between February 2010 and December 2013).^41^ Both groups received detachable acrylic prostheses with cast metal frameworks, completely supported, stabilized and retained by SynCone telescopic abutments.

None of the four trials used grafting or other ridge augmentation procedures before implant insertion. Participants in CL or EL wore conventional complete dentures relined with soft materials before insertion of definitive prostheses.

### Methodological quality of the trials

**Figure 2:**
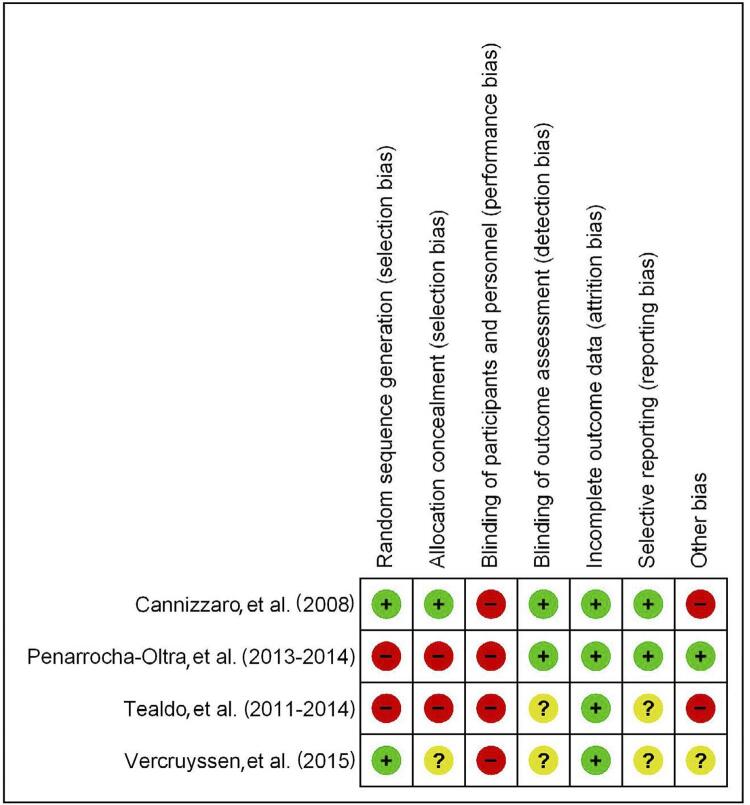
Risk of bias summary for included studies: evaluations on risk of bias concerning each potential source and type of bias. A + signifies that the corresponding approach to minimize bias was probably done (adequately described) for a given study, whereas a - discloses an evident limitation in controlling bias. A question mark underscores that the study provides insufficient description for judging a given approach as adequate or not

All the four trials showed some potential source of bias classified as “high risk”. Figure 2 summarizes the quality assessment of the four included trials. Appendix 4 details the methodological quality assessment of individual trials.

Sequence generation was adequate for Canizzaro, et al.^38^ (2008) and Vercruyssen, et al.^41^ (2016), whereas only the first was explicit regarding the use of allocation concealment. The other CCTs [Peñarocha-Oltra, et al.^13 , 14^ (2013, 2014) and Tealdo, et al.^39 , 40^ (2011, 2014)] were preference trials; therefore, they were classified at high risk for selection bias-related criteria.

All trials had high risk for performance bias as a limitation — patients cannot be treated blindly, and no study described any approach to prepare prostheses in a way that could mitigate this source of bias. Two trials performed a blind outcome assessment,^13 , 14 , 38^ whereas other two study reports provided no data on blinding for eligible outcomes.^39 - 41^

Incomplete outcome data was a minor concern for the four trials. Two trials reported a comprehensive series of outcomes in a way that consistently leads to “low risk” classification for selective reporting.^13 , 14 , 38^ There was no study protocol for any of the studies, thus selective reporting was unclear for the other two trials.^39 - 41^

Finally, other potential sources of bias included between-group imbalances regarding: (1) the final prosthesis provided by one of the studies, i.e. Toronto-type acrylic prostheses, IL: 4 (27%); EL: 9 (60%) participants;^38^ and (2) number of implants, i.e. IL received less implants/maxillary denture than the CL group.^39 , 40^ One of the preference trials is very unlikely affected by other biases,^13 , 14^ and we could not determine whether sponsorship would influence results of an RCT.^41^

### Effect of interventions

Table 2 summarizes the main findings of the four included trials, according to each outcome.

**Table 2:** Summary of outcome data from included studies (NR: not reported)

Study	Patient satisfaction	Implant failure and survival rate (SR)	Peri-implant bone level (mm)*	Maxillary Prostheses, SR	Prosthetic complications**
**IL versus EL:**					
Cannizzaro, et al.^38^ (2008)	"Completely satisfied" answer, 12 mo. (n):	Failed Implants, 12 mo. (n/total):	- Baseline, IL: 0.1(0.1); EL: 0.1 (0.1)	NR	IL, Total: 8
	- IL: 11 (73%)	- IL: 1/90, SR = 98.8%	- 12 mo., IL: 0.7 (0.2); EL: 0.8 (0.2)		- Ulcers by provisional: 1
	- EL: 5 (33%)	- EL: 3/87, SR = 96.5%			- Fractured provisional: 2
					- Fractured final prosthesis: 1
					- Masticatory/TMJ problem: 2
					- Peri-implant complications: 2
					EL, total: 5
					- Fractured provisional: 2
					- Masticatory/TMJ problem: 1
					- Peri-implant complications: 1
					- Esthetics: 1

**IL versus CL:**					

Peñarrocha-Oltra, et al.^13,14^ (2013, 2014)	100-mm VAS, mean (SD) for IL and CL:	Failed Implants, 12 mo. (n/total):	Baseline, both groups: 0.2	- 100% both arms (12 mo.)	- IL, Total: 8 (4 loose screws, 1 tooth fracture, 3 mucositis)
	- Baseline: 45 (18) and 48 (17)	- IL: 3/94, SR = 96.8%	-12 mo., IL: 0.6 (0.2); CL: 0.6 (0.3)		
	- 3 mo.: 85 (11) and 50 (13)	- CL: 1/99, SR = 99.0%			- CL, Total: 8 (3 mucositis; 5 ulcers)
	- 12 mo.: 90 (7) and 90 (10)				
Tealdo, et al.^39,40^ (2011, 2014)	NR	Failed Implants, 12 mo. (n/total):	Baseline, both groups: 0.5	- 100% both arms (72 mo.)	- IL, Total: 9 (4 minor fractures, 2 major fractures, 3 loose screws)
		- IL: 10/163, SR = 93.9%	- 12 mo., IL: 1.3 (0.8); CL: 1.9 (0.8)		
		- CL: 4/97, SR = 95.9%	- 24 mo., IL: 1.5 (0.9); CL: 2.2 (0.9)	- Success rate: IL: 82.4%, CL: 73.3% (72 mo.)	- CL, Total: 9 (3 minor fractures; 1 major fracture; 5 loose screws)
		No failed implant between 12 and 72 mo.	- 36 mo., IL: 1.6 (0.9); CL: 2.3 (1.1)		
			- 72 mo., IL: 1.6 (1.2); CL: 2.4 (1.4)		
Vercruyssen, et al.^41^ (2016)	NR	Failed Implants, 3 mo. (n/total):	NR	NR	NR
		- IL: 0/42, SR = 100%			
		- DL: 1/48, SR = 97.9%			

* Distance between most coronal portion implant-bone contact area and coronal margin of implant collar; ** At the longest follow-up period/total n comprises prosthetic complications + others

#### Primary outcomes

IL versus EL: Canizzaro, el al.^38^ (2008) performed a single assessment at the 12-month follow-up by asking whether patients were satisfied with overall treatment, indicated on a 5-point Likert scale. The RR of having participants more satisfied with IL after 12 months was 2.20 (95%CI: 1.01 to 4.79).

IL versus CL: Peñarrocha-Oltra, et al.^13 , 14^ (2013, 2014) quantified overall patient satisfaction on a 100-mm VAS. The average value after 3 months was 35 mm higher for the IL arm (95%CI: 26 to 44 mm). Such difference recedes after 12-months follow-up (mean difference: 0; 95%CI: -6 to 6 mm).

#### Secondary outcomes

A single trial reported specific patient satisfaction items, by questions answered on a 100 mm-VAS.^13 , 14^ At 3 months, mean differences between IL and CL for separate items were (in mm; positive values favor IL): esthetics: 20, 95%CI: 9 to 32; chewing: 48, 95%CI: 33 to 63; speech: 25, 95%CI: 12 to 38; comfort: 53, 95%CI: 39 to 67; self-esteem: 33, 95%CI: 21 to 45; ease of cleaning: -6, 95%CI: -19 to 7; treatment duration: 40, 95%CI: 32 to 48. At 12 mo., mean differences were: esthetics: -9, 95%CI: -17 to -1; chewing: 1, 95%CI: -6 to 8; speech: 3, 95%CI: -4 to 10; comfort: -2, 95%CI: -8 to 4; self-esteem: 0, 95%CI: -7 to 7; ease of cleaning: -2, 95%CI: -12 to 8; treatment duration: 8, 95%CI: -5 to 21.

The total n of studied implants were: (IL) 299; (CL) 244; and (EL) 87. There was no evidence that implant survival was different with IL or other protocols. Data comparing IL to EL comes from a single trial^38^ and observed a 1-year RR of 0.32 (95%CI: 0.03 to 3.04) with IL. A single study comparing IL to CL^41^ observed a RR of 0.38 (95%CI: 0.02 to 9.08) up to 3 months, and two heterogeneous trials found similar RR after 12 months: 3.16 (95%CI: 0.33 to 29.84);^13 , 14^ 1.49 (95%CI: 0.48 to 4.61).^39 , 40^ The latter study observed no implant failure between 12 and 72 months.

Results show no evident difference regarding peri-implant bone level observed by radiographs comparing IL to EL and CL. Compared to EL, mean changes after 12 mo. were similar for IL:^38^ – mean difference: 0.07 mm (95%CI: -0.10 to 0.24). Mean differences in bone level between IL and CL were very small at 12 months, ranging from 0.0 mm (95%CI: -0.18, 0.18)^13 , 14^ to -0.60 mm (95%CI: -1.10, -0.10).^39 , 40^ The latter difference was significant, and reached -0.80 mm after 72 months (95%CI: -1.64 to 0.04).

Finally, it seems that the IL groups had higher cumulative incidence of prosthetic complications compared to the other groups on the short term and one year-long term. Cumulative rates of mechanical failures for separate studies were: Peñarrocha-Oltra, et al.^13 , 14^ (2013, 2014), IL: 62.5%; CL: 0%; Tealdo, et al.^39 , 40^ (2011, 2014), IL: 50%; CL: 50%; Canizzaro, et al.^38^ (2008), IL: 50%; EL: 25%. Most of those failures occurred with provisional prostheses in IL.

## Discussion

Despite the impact of IL for the management of edentulous maxillae, this review included a small number of CCTs. A recent growing interest in the literature on the subject is evident given the year of the oldest included report (i.e. 2008).^38^ This contrasts with the wide proportion of observational studies on IL found in 2005,^42^ thus suggesting a recent shift towards CCTs.

Comparisons between IL and comparators show that patients may be more satisfied when they receive a functional fixed denture, regardless of when. Evidence is minor, but IL was more satisfying than EL in a single RCT after 1 year.^38^ However, that trial evaluated satisfaction as a secondary outcome and performed a simple assessment. An imbalance in the types of prostheses delivered to the two groups may also have contributed to post-treatment differences. Therefore, as tempting it is to suggest a long-term effect of IL on patient satisfaction, this finding should be interpreted carefully. A trial comparing IL to CL showed similar treatment effect after 3 months;^13 , 14^ this is intuitive, given that participants still had relined conventional dentures in the CL group. Results for satisfaction are similar at 12 months though, suggesting that results may not differ at that point. Patients may get used with existing fixed dentures and provide similar responses after few months. In other words, patients may undergo a response shift and reach similar perception of received prostheses regardless of initial experiences.^43^

In general, findings suggest that IL is effective compared to EL and CL, although evidence is not enough for solid clinical recommendations. Clinician-reported outcomes show no evident difference in survival rates for implants and prostheses. Failures tend to be quite rare. Other complications show no difference, although a synthesis of the four trials was unviable. Bone loss was not different when IL was compared to other protocols. A trial observed a lower complication rate with IL compared to CL, possibly caused by different prosthetic configurations/n of implants rather than the loading protocol itself.

All studies provided treatment with standard dental implants, thus evidence from CCTs is absent for zygomatic implants. Their potential safety and effectiveness make them a very interesting subject for future trials, as found by observational studies.^10 , 44 , 45^

Trial participants represent average edentulous patients regarding age and gender, who can receive standard implants without ridge augmentation. No data can be extrapolated to patients with severely atrophied maxillae, who may need bone augmentation procedures (e.g. onlay bone grafts and sinus lifting) or zygomatic implants. Furthermore, most inclusions refer to IL versus CL, with a single trial with EL as a comparator.

Three out of the four included trials were conducted at university clinics. This may not be a major issue given that specialists normally provide tested interventions. However, it is arguable whether results are exactly the same expect for routine patients without research involvement. For instance, potential participants may refrain to participate given potential concerns regarding randomization.^32^ The inclusion of preference trials may mitigate such issues, by rendering study participants closer to real patients, with freedom to deliberate on which treatment they will receive.^46 , 47^

The paucity of studies makes any assumption regarding specific clinical conditions unclear. For example, one cannot infer whether different results are expected because of different occlusal schemes or antagonist arch. The same could not be done for certain adverse conditions that could contra-indicate IL, e.g. severe parafunction, smoking, and high risk of periodontal disease.^48 , 49^

In summary, all included studies could be classified as high risk of bias for varying reasons. Amongst design-related issues, the inclusion of preference trials deserves comments, given their important drawback: higher risk of selection bias.^50^ Those trials cannot implement sequence generation methods able to minimize selection bias. Blinding also was a major limitation, given that blinding the participants and care providers is not possible for the tested comparisons. In general, studies were careful when reporting the numbers of non-adherent participants.

Given the long-lasting recommendation of trial registration and contemporaneity of included trials, the absence of published protocols was surprising. Trial registration has been a persisting recommendation of guidelines for trial protocols^51^ and final reports.^52^

One of the main limitation of this review is the low number of included studies. A scarcity of RCTs was foreseeable and approached by widening eligibility criteria to preference trials and other non-randomized CCTs. However, even this approach resulted in a considerably low number of trials. Summed to the finding of only two trials reporting our primary outcomes, this review is further limited to the non-comparability of different questionnaires for patient satisfaction. Studies were also underpowered (modest sample sizes) for categorical outcomes. Major clinical heterogeneity also proscribes meta-analysis and thus contributes to the power-related issue. Our search strategy attempted to approach a wide series of potential sources for better sensitivity. Although we were initially limited to reports written in English, our search did not found non-English studies. Thus, language cannot be considered as a limitation of this review.

This systematic review innovates by its patient-centered focus, which is uncommon in other reviews. However, it is notable that previous reviews found akin results for clinician/disease-centered outcomes. Esposito, et al.^15^ (2013) found similar survival and success rates for different loading methods. That review only considered clinical performance, and missed four of our six included reports given its last update timing.^15^ Finally, we extended the eligibility criteria to include preference trials, different from that review. Further three recent systematic reviews on IL’s clinical outcomes^53 - 55^ found a single CCT.^38^ Other reviews did not find CCTs comparing immediately loaded zygomatic implants to other loading protocols on similar fixtures either.^56 , 57^

Future trials are fundamental to compare IL to other loading approaches in the edentulous maxilla, and should consider zygomatic fixtures. Given that many patients can cope well with maxillary conventional dentures, and that the cost/complexity of IL may be quite high, recruitment in such trials can be quite slow. Multicenter RCTs can overcome those issues and timely reach a good sample size. Such tentative trial(s) should use standardized tools for outcome assessment at several recall visits —baseline up to at least 12 months, but focusing on short-term follow-up. Focus on patient-reported outcomes is imperative, given their fundamental role for clinical guidelines/recommendations.^17^ The use of reporting guidelines (e.g. SPIRIT and CONSORT) will lead to more transparent and comprehensive research methods, as well as trial registration in public databases (e.g. clinicaltrials.gov).

## Conclusions

This review found modest evidence on the comparative performance of IL versus other loading regimens (CL and EL) for providing fully implant-supported maxillary dental prostheses. A limited number of trials suggest that patient satisfaction may be at least as good with IL, and show no major discrepancies regarding clinical complications.

The selection of IL instead of CL or EL must rest on solid practitioner’s skills to provide such treatment and patient preferences. Evidence supports effective use of IL for fixed full prostheses on standard implants, given that no augmentation method is used. Patients seem at least as satisfied with IL, and clinical complications may be comparable. Comparative evidence on cases with unfavorable clinical features remains scant.

## Appendix 2

**Table:** Systematic review search strategy in Medline (Ovid), composed by terms representing our interventions of interest, eligible participants (population), and comparators. MeSH terms and free text words were combined for the search using Boolean operators

	Search date: 17/01/2019 Note: CL was not included because it is anticipated to be the common comparator.
Intervention and Comparison	1. exp Dental Implants/ 2. exp Dental Implantation/ 3. exp Dental Prosthesis, Implant-Supported/ 4. ((osseintegrat* adj3 implant$) and (dental* or oral*)).ti,ab,kf. 5. (((overdenture* or crown* or bridge* or prosthes?s or restoration*) adj5 (dental* or oral*)) and implant*).ti,ab,kf. 6. "implant supported dental prosthesis".ti,ab,kf. 7. ("blade implant*" and (dental* or oral*)).ti,ab,kf. 8. ((endosseous adj5 implant*) and (dental* or oral*)).ti,ab,kf. 9. ((dent* or oral* or zygomatic or axial or tilted) adj5 implant*).ti,ab,kf. 10. or/1-9 11. ((early or immediate*) adj3 (loaded or loading or restoration or rehabilitat*)).ti,ab,kf.
Population	12. exp Maxilla/ 13. maxilla*.ti,ab,kf. 14. ((zygomatic or alveolar or palatine) adj process*).ti,ab,kf. 15. or/12-14 16. exp Mandible/ 17. (mandible* or mandibular*).ti,ab,kf. 18. Jaw, Edentulous/ 19. (edentulous* or edentate or edentulism).ti,ab,kf. 20. 18 or 19
Outcomes	Not included
Filters	None
Final search	21. 10 and 11 and 15 and 20 22. 11 and 15 and 20

## Appendix 3

**Table:** Excluded studies after full-text assessment and reasons

Study	Reason
Agnini, et al.^24^(2014)	No comparator group
Aires and Berger^25^(2002)	Not a clinical trial
Alves, et al.^26^(2010)	Not a clinical trial
Aparicio, et al.^27^(2010)	No comparator group
Busenlechner, et al.^28,29^(2016)	Not a clinical trial
Babbush, et al.^30^(2013)	Not a clinical trial, no comparator group
Calandriello and Tomatis^31^(2005)	No comparator group
Esposito, et al.^32^(2016); Mitsias, et al.^33^(2018)	Participants cannot be considered
Esposito, et al.^34^(2018)	Comparator cannot be considered
Nordin, et al.^35,36^(2004, 2007)	Not a clinical trial, intervention cannot be considered
Zhou, et al.^37^(2009)	Participants cannot be considered

## Appendix 4

**Table:** Detailed risk of bias assessment according to the Cochrane Risk of Bias tool. Author’s judgement refers to the classification scale for risk of bias (low/unclear/high); Support for judgment will contain a critical appraisal leading to each closed-ended answer, including quotes that led to judgement

STUDY ID: Canizzaro, et al.^**38**^ (2008) [IL compared to EL]		
Source of Bias	Judgment	Support for judgement
Random sequence generation (selection bias)	Low	Quote: "A computer generated restricted randomization list was used to create two groups with equal numbers of patients by one of the authors, who was not involved in patient recruitment or treatment and had access to the randomization list stored in a password-protected portable computer"
Allocation concealment (selection bias)	Low	Quote: "After all implants were inserted..., the envelope containing the randomization code was opened and the operator knew whether the patient would have the implants immediately loaded or loaded after 2 months"
		Quote: "The randomized codes were enclosed in sequentially numbered, identical, opaque, sealed envelopes. Envelopes were opened sequentially only after all the implants were inserted, therefore treatment allocation was concealed to the investigator in charge of enrolling and treating the patients included in the trial."
Blinding of participants and personnel (performance bias)	High	N/A for participants and personnel.
Blinding of outcome assessment (detection bias)	Low	Quote: "Independent dentists who were not aware of patient allocation evaluated implant stability, including ISQ values ... and marginal bone levels changes..."
		Quote: "A biostatistician... analyzed the data, without knowing the group allocation."
Incomplete outcome data (attrition bias)	Low	No dropout or loss to follow-up, all participants were included in the statistical analysis.
Selective reporting (reporting bias)	Low	A comprehensive set of clinical outcomes was reported, as well as patient satisfaction -- unlikely selective reporting.
Other bias	High	Type of prosthetic treatment is imbalanced in the two groups, with possible influence on patient satisfaction: Toronto-type acrylic prostheses, IL: 4 (27%); EL: 9 (60%); other participants received PFM (less provided needed for the upper lip, i.e. better ridge anatomy).
		Recruitment happened in clinical practice (probably as part of routine care), unlikely conflict of interest.
		Quote: "Patients were recruited and treated in one Italian private practice" and "No commercial support of any form has been received by the investigators".
**STUDY ID: Peñarrocha-Oltra, et al.** ^**13,14**^ **(2013, 2014) [IL compared to CL]**		
**Source of Bias**	**Judgment**	**Support for judgement**
Random sequence generation (selection bias)	High	Non-randomized sequence
		Quote: “A clinical prospective controlled nonrandomized study was performed at the Oral Surgery Unit”.
Allocation concealment (selection bias)	High	Open allocation sequence
		Quote: “15 consecutive patients fulfilling the se- lection criteria were treated following a conventional loading protocol (control group) until July 2009… The next 15 consecutive patients fulfilling the inclusion criteria were, therefore, treated with this protocol (test group).”
Blinding of participants and personnel (performance bias)	High	N/A for participants and personnel.
Blinding of outcome assessment (detection bias)	Low	Blind outcome assessor
		Quote: “All data were collected by a single trained clinician (DP), who was not the surgeon or the prosthodontist, following a pre-established protocol.”
Incomplete outcome data (attrition bias)	Low	One drop-out (test group); reason unlikely to be related to intervention. Some unloaded implants in both groups, with similar numbers and reasons and no change in assigned intervention.
		Quote: “One patient belonging to the test group failed to attend the scheduled recall visits because of personal reasons and was excluded from the study.”
		“Sixteen implants—nine in the test group and seven in the control group, all of which were placed in molar regions—did not achieve the minimum insertion torque of 35 Ncm and were excluded from analysis, left submerged, and loaded conventionally.”
Selective reporting (reporting bias)	Low	Most implant and prosthetic success criteria were reported, including adverse events.
Other bias	Low	Unlikely bias from other sources.
**STUDY ID: Vercruyssen, et al.** ^**41**^ **(2016) (IL compared to CL)**		
**Source of Bias**	**Judgment**	**Support for judgement**
Random sequence generation (selection bias)	Low	Possibly done, but no explanation of method.
		Quote: “For the allocation, a computerized random number generator was used.”
Allocation concealment (selection bias)	Unclear	No detail on how the random numbers were applied (e.g. an open list or generated immediately before each intervention).
Blinding of participants and personnel (performance bias)	High	N/A for participants and personnel.
Blinding of outcome assessment (detection bias)	Unclear	No specific information regarding outcome of interest.
Incomplete outcome data (attrition bias)	Low	Quote: “one implant from the delayed treatment group was lost before prosthesis installment due to non-integration.”
Selective reporting (reporting bias)	Unclear	Specific set of clinician-reported outcomes and short-term patient-reported outcome assessment (pain/discomfort and general health-related quality of life). No data on key outcomes used in oral implantology.
Other bias	Unclear	Oral implants were delivered free of charge by DENTSPLY Implants (Molndal, Sweden). Stereolithographic guides were delivered free of charge by the Materialise Dental Company (Leuven, Belgium).
**STUDY ID: Tealdo, et al.** ^**39,40**^ **(2011, 2014) (IL compared to CL)**		
**Source of Bias**	**Judgment**	**Support for judgement**
Random sequence generation (selection bias)	High	Participants were allocated according to their preferences to one of the interventions.
Allocation concealment (selection bias)	High	Open allocation.
		Quote: “The patients in the test group were selected for treatment with the immediate loading protocol because of both their expectations and demand for immediate, fixed implant prostheses; they sought to avoid the use of a transitional complete denture. On the other hand, the patients in the control group were willing to accept wearing a complete denture for a short time interval, and this cohort was composed of older patients relative to the test group.”
Blinding of participants and personnel (performance bias)	High	N/A for participants and personnel.
Blinding of outcome assessment (detection bias)	Unclear	No blinding mentioned.
		Quote: “Subjects were seen by a dental hygienist every 4 months for the first year. At each follow-up visit, prostheses were removed and implants and abutments were evaluated individually for tenderness, swelling, and mobility.”
Incomplete outcome data (attrition bias)	Low	Low number of dropouts (n=1/group), but reasons are unclear. Few losses due to reasons unlikely associated with protocol. Quote: “At the 6-year follow-up, 2 patients had dropped out. One patient with 4 implants in the test group died, and 1 patient in the control group with 7 implants relocated.
Selective reporting (reporting bias)	Unclear	Study focuses on implants’ clinical performance, and do not report relevant patient-reported outcomes.
Other bias	High	Different number of implants may confound the effect of immediate versus delayed loading.

## References

[1] Cunha-Cruz J, Hujoel PP, Nadanovsky P (2007). Secular trends in socio-economic disparities in edentulism: USA, 1972-2001. J Dent Res.

[2] Emami E, Souza RF, Kabawat M, Feine JS (2013). The impact of edentulism on oral and general health. Int J Dent.

[3] Cunha TR, Della Vecchia MP, Regis RR, Ribeiro AB, Muglia VA, Mestriner W (2013). A randomised trial on simplified and conventional methods for complete denture fabrication: masticatory performance and ability. J Dent.

[4] Michael CG, Javid NS, Colaizzi FA, Gibbs CH (1990). Biting strength and chewing forces in complete denture wearers. J Prosthet Dent.

[5] Trulsson U, Engstrand P, Berggren U, Nannmark U, Brånemark PI (2002). Edentulousness and oral rehabilitation: experiences from the patients’ perspective. Eur J Oral Sci.

[6] Feine JS, Grandmont P, Boudrias P, Brien N, LaMarche C, Taché R (1994). Within-subject comparisons of implant-supported mandibular prostheses: choice of prosthesis. J Dent Res.

[7] Emami E, Michaud PL, Sallaleh I, Feine JS (2014). Implant-assisted complete prostheses. Periodontology 2000.

[8] Thomason JM, Kelly SA, Bendkowski A, Ellis JS (2012). Two implant retained overdentures: a review of the literature supporting the McGill and York consensus statements. J Dent.

[9] Preoteasa E, Meleşcanu-Imre M, Preoteasa CT, Marin M, Lerner H (2010). Aspects of oral morphology as decision factors in mini-implant supported overdenture. Rom J Morphol Embryol.

[10] Coppedê A, Mayo T, Sá Zamperlini M, Amorin R, Pádua A, Shibli JA (2017). Three-year clinical prospective follow-up of extrasinus zygomatic implants for the rehabilitation of the atrophic maxilla. Clin Implant Dent Relat Res.

[11] Albuquerque RF, Lund JP, Tang L, Larivée J, Grandmont P, Gauthier G (2000). Within-subject comparison of maxillary long-bar implant-retained prostheses with and without palatal coverage: patient-based outcomes. Clin Oral Implants Res.

[12] Hopp M, Araújo Nobre M, Maló P (2017). Comparison of marginal bone loss and implant success between axial and tilted implants in maxillary All-on-4 treatment concept rehabilitations after 5 years of follow-up. Clin Implant Dent Relat Res.

[13] Peñarrocha-Oltra D, Covani U, Aparicio A, Ata-Ali J, Peñarrocha-Diago M, Peñarrocha-Diago M (2013). Immediate versus conventional loading for the maxilla with implants placed into fresh and healed extraction sites to support a full-arch fixed prosthesis: nonrandomized controlled clinical study. Int J Oral Maxillofac Implants.

[14] Peñarrocha-Oltra D, Peñarrocha-Diago M, Canullo L, Covani U, Peñarrocha M (2014). Patient-reported outcomes of immediate versus conventional loading with fixed full-arch prostheses in the maxilla: a nonrandomized controlled prospective study. Int J Oral Maxillofac Implants.

[15] Esposito M, Grusovin MG, Maghaireh H, Worthington HV (2013). Interventions for replacing missing teeth: different times for loading dental implants. Cochrane Database Syst Rev.

[16] Souza RF, Ahmadi M, Ribeiro AB, Emami E (2014). Focusing on outcomes and methods in removable prosthodontics trials: a systematic review. Clin Oral Implants Res.

[17] Ebell MH, Siwek J, Weiss BD, Woolf SH, Susman J, Ewigman B (2004). Strength of recommendation taxonomy (SORT): a patient-centered approach to grading evidence in the medical literature. Am Fam Physician.

[18] Moher D, Liberati A, Tetzlaff J, Altman DG, PRISMA Group (2009). Preferred reporting items for systematic reviews and meta-analyses: the PRISMA statement. PLoS Med.

[19] Abdunabi A, Morris M, Abi Nader S, Souza R (2018). Impact of immediate loaded implant-supported fixed dental prostheses on edentulous maxillae: a systematic review.

[20] Tonetti M, Palmer R, Working Group 2 of the VIII European Workshop on Periodontology (2012). Clinical research in implant dentistry: study design, reporting and outcome measurements: consensus report of Working Group 2 of the VIII European Workshop on Periodontology. J Clin Periodontol.

[21] Higgins JP, Altman DG, Gøtzsche PC, Jüni P, Moher D, Oxman AD (2011). The Cochrane Collaboration’s tool for assessing risk of bias in randomised trials. BMJ.

[22] Higgins JP, Green S (2018). Cochrane Handbook for Systematic Reviews of Interventions.

[23] Egger M, Davey Smith G, Schneider M, Minder C (1997). Bias in meta-analysis detected by a simple, graphical test. BMJ.

[24] Agnini A, Agnini AM, Romeo D, Chiesi M, Pariente L, Stappert CF (2014). Clinical investigation on axial versus tilted implants for immediate fixed rehabilitation of edentulous arches: preliminary results of a single cohort study. Clin Implant Dent Relat Res.

[25] Aires I, Berger J (2002). Immediate placement in extraction sites followed by immediate loading: a pilot study and case presentation. Implant Dent.

[26] Alves CC, Correia AR, Neves M (2010). Immediate implants and immediate loading in periodontally compromised patients-a 3-year prospective clinical study. Int J Periodontics Restorative Dent.

[27] Aparicio C, Ouazzani W, Aparicio A, Fortes V, Muela R, Pascual A (2010). Immediate/Early loading of zygomatic implants: clinical experiences after 2 to 5 years of follow-up. Clin Implant Dent Relat Res.

[28] Busenlechner D, Mailath-Pokorny G, Haas R, Fürhauser R, Eder C, Pommer B (2016). Graftless full-arch implant rehabilitation with interantral implants and immediate or delayed loading-part II: transition from the failing maxillary dentition. Int J Oral Maxillofac Implants.

[29] Busenlechner D, Mailath-Pokorny G, Haas R, Fürhauser R, Eder C, Pommer B (2016). Graftless full-arch implant rehabilitation with interantral implants and immediate or delayed loading-part I: reconstruction of the edentulous maxilla. Int J Oral Maxillofac Implants.

[30] Babbush CA, Kanawati A, Brokloff J (2013). A new approach to the All-on-Four treatment concept using narrow platform NobelActive implants. J Oral Implantol.

[31] Calandriello R, Tomatis M (2005). Simplified treatment of the atrophic posterior maxilla via immediate/early function and tilted implants: a prospective 1-year clinical study. Clin Implant Dent Relat Res.

[32] Esposito M, Siormpas K, Mitsias M, Bechara S, Trullenque-Eriksson A, Pistilli R (2016). Immediate, early (6 weeks) and delayed loading (3 months) of single implants: 4-month post-loading from a multicenter pragmatic randomised controlled trial. Eur J Oral Implantol.

[33] Mitsias M, Siormpas K, Pistilli V, Trullenque-Eriksson A, Esposito M (2018). Immediate, early (6 weeks) and delayed loading (3 months) of single, partial and full fixed implant supported prostheses: 1-year post-loading data from a multicentre randomised controlled trial. Eur J Oral Implantol.

[34] Esposito M, Davó R, Marti-Pages C, Ferrer-Fuentes A, Barausse C, Pistilli R (2018). Immediately loaded zygomatic implants vs conventional dental implants in augmented atrophic maxillae: 4 months post-loading results from a multicentre randomised controlled trial. Eur J Oral Implantol.

[35] Nordin T, Graf J, Frykholm A, Helldén L (2007). Early functional loading of sand-blasted and acid-etched (SLA) Straumann implants following immediate placement in maxillary extraction sockets. Clinical and radiographic result. Clin Oral Implants Res.

[36] Nordin T, Nilsson R, Frykholm A, Hallman M (2004). A 3-arm study of early loading of rough-surfaced implants in the completely edentulous maxilla and in the edentulous posterior maxilla and mandible: results after 1 year of loading. Int J Oral Maxillofac Implants.

[37] Zhou W, Han C, Yunming L, Li D, Song Y, Zhao Y (2009). Is the osseointegration of immediately and delayed loaded implants the same? Comparison of the implant stability during a 3-month healing period in a prospective study. Clin Oral Implants Res.

[38] Cannizzaro G, Torchio C, Leone M, Esposito M (2008). Immediate versus early loading of flapless-placed implants supporting maxillary full-arch prostheses: a randomised controlled clinical trial. Eur J Oral Implantol.

[39] Tealdo T, Bevilacqua M, Menini M, Pera F, Ravera G, Drago C (2011). Immediate versus delayed loading of dental implants in edentulous maxillae: a 36-month prospective study. Int J Prosthodont.

[40] Tealdo T, Menini M, Bevilacqua M, Pera F, Pesce P, Signori A (2014). Immediate versus delayed loading of dental implants in edentulous patients’ maxillae: a 6-year prospective study. Int J Prosthodont.

[41] Vercruyssen M, Cox C, Naert I, Jacobs R, Teughels W, Quirynen M (2016). Accuracy and patient-centered outcome variables in guided implant surgery: a RCT comparing immediate with delayed loading. Clin Oral Implants Res.

[42] Attard NJ, Zarb GA (2005). Immediate and early implant loading protocols: a literature review of clinical studies. J Prosthet Dent.

[43] Ring L, Höfer S, Heuston F, Harris D, O’Boyle CA (2005). Response shift masks the treatment impact on patient reported outcomes (PROs): the example of individual quality of life in edentulous patients. Health Qual Life Outcomes.

[44] Bedrossian E, Rangert B, Stumpel L, Indresano T (2006). Immediate function with the zygomatic implant: a graftless solution for the patient with mild to advanced atrophy of the maxilla. Int J Oral Maxillofac Implants.

[45] Hirsch JM, Ohrnell LO, Henry PJ, Andreasson L, Brånemark PI, Chiapasco M (2004). A clinical evaluation of the zygoma fixture: one year of follow-up at 16 clinics. J Oral Maxillofac Surg.

[46] Marcus SM, Stuart EA, Wang P, Shadish WR, Steiner PM (2012). Estimating the causal effect of randomization versus treatment preference in a doubly randomized preference trial. Psychol Methods.

[47] Sidani S, Fox M, Epstein DR (2017). Contribution of treatment acceptability to acceptance of randomization: an exploration. J Eval Clin Pract.

[48] Bahat O, Sullivan RM (2010). Parameters for successful implant integration revisited part II: algorithm for immediate loading diagnostic factors. Clin Implant Dent Relat Res.

[49] Bahat O, Sullivan RM (2010). Parameters for successful implant integration revisited part I: immediate loading considered in light of the original prerequisites for osseointegration. Clin Implant Dent Relat Res.

[50] Archer S, Forshaw MJ (2015). Using a randomised controlled trial (RCT) methodology in CAM research with gynaecological cancer patients: a commentary on the perks and pitfalls. Complement Ther Clin Pract.

[51] Chan AW, Tetzlaff JM, Gøtzsche PC, Altman DG, Mann H, Berlin JA (2013). SPIRIT 2013 explanation and elaboration: guidance for protocols of clinical trials. BMJ.

[52] Schulz KF, Altman DG, Moher D, CONSORT Group (2010). CONSORT 2010 statement: updated guidelines for reporting parallel group randomised trials. PLoS Med.

[53] Gallucci GO, Morton D, Weber HP (2009). Loading protocols for dental implants in edentulous patients. Int J Oral Maxillofac Implants.

[54] Papaspyridakos P, Chen CJ, Chuang SK, Weber HP (2014). Implant loading protocols for edentulous patients with fixed prostheses: a systematic review and meta-analysis. Int J Oral Maxillofac Implants.

[55] Strub JR, Jurdzik BA, Tuna T (2012). Prognosis of immediately loaded implants and their restorations: a systematic literature review. J Oral Rehabil.

[56] Candel-Marti E, Carrillo-Garcia C, Peñarrocha-Oltra D, Peñarrocha-Diago M (2012). Rehabilitation of atrophic posterior maxilla with zygomatic implants: review. J Oral Implantol.

[57] Tuminelli FJ, Walter LR, Neugarten J, Bedrossian E (2017). Immediate loading of zygomatic implants: a systematic review of implant survival, prosthesis survival and potential complications. Eur J Oral Implantol.

